# The effects of an earthquake experienced during pregnancy and perinatal outcomes

**DOI:** 10.1590/1806-9282.20241331

**Published:** 2025-03-17

**Authors:** Asibe Özkan, Melike Dissiz, Zehra Acar, Rojjin Mamuk

**Affiliations:** 1 Health Science University, Hamidiye Faculty of Nursing – İstanbul, Turkey.; 2 Eastern Mediterranean University, Faculty of Health Sciences – Famagusta, Cyprus.

**Keywords:** Disasters, Earthquake, Perinatal care, Perinatal nursing

## Abstract

**OBJECTIVE:**

The aim of this study was to evaluate the effects of an earthquake experienced during pregnancy on maternal stress, depression, and perinatal outcomes.

**METHODS:**

This descriptive–comparative study was conducted between April and May 2023 in four state hospitals affiliated to the Ministry of Health with 82 mothers (affected by the earthquake=41, not affected by the earthquake=41) who gave birth. Data were obtained with the Personal Information Form, Edinburgh Postpartum Depression Scale, and Traumatic Stress Symptom Checklist.

**RESULTS:**

The mean age of the mothers who were earthquake affected in the study was 27.63±5.62. It was determined that earthquake-affected mothers gave birth prematurely and the birth weight of their infants was lower (p<0.05). It was determined that all of the earthquake-affected mothers experienced more stress according to the Traumatic Stress Symptom Checklist scale cut-off score (>25) and 73.2% of them showed more depressive symptoms according to the Edinburgh Postpartum Depression Scale cut-off score (>13) (p<0.05). According to multivariate regression analysis, early gestational age and depressive symptoms were found to be risk factors for posttraumatic stress in mothers affected by the earthquake (p<0.05).

**CONCLUSION:**

The study concluded that the earthquake-affected mothers showed depressive symptoms at least 6 weeks after giving birth, and all had increased levels of posttraumatic stress.

## INTRODUCTION

The two major earthquakes in Turkey, which affected more than 9.1 million people and killed 50,000 people, are among the biggest disasters of the last century^1^. Women and children are more vulnerable to these disasters and have been shown to be more prone to post-disaster psychopathology than men^2-4^. It would not be surprising that natural disasters such as earthquakes, which expose every individual in society to extreme stress, affect the mental health of pregnant women, and thus prenatal outcomes^2,5^. Negative outcomes such as posttraumatic stress disorder, depression, and suicide have been reported, especially in pregnant and puerperant women after earthquakes^4^.

It has also been shown that exposure to stressful events such as disasters may also affect the intrauterine environment and fetal development, leading to low birth weight, lower APGAR scores, a smaller head circumference, and higher preterm birth rates in the baby^4,5^. In a study conducted to determine the mental health of women who became mothers during the Great East Japan earthquake, it was determined that the majority of women experienced depression^5^.

In current studies, little is known about the effect of earthquakes on pregnant and postpartum women. For this reason, after the severe earthquakes that occurred one after another and had a large-scale destructive impact on Turkey, it is thought that determining their effects on pregnant and puerperal women will contribute to filling this important gap in the literature. From this point of view, the aim of this study was to compare the effects of earthquakes experienced during pregnancy on maternal stress and depression and perinatal outcomes in mothers affected and unaffected by earthquakes.

## METHODS

### Type, place, and time of the study

The study was planned and implemented in the comparative descriptive type. The data for the study were obtained from four public hospitals affiliated with the Ministry of Health located within the provincial borders of Istanbul between April and May 2023.

### Participants and procedure

A total of 41 earthquake-affected mothers who were referred from the earthquake zone to give birth in four different hospitals in Istanbul, gave birth in these four hospitals, were at least 6 weeks postpartum (to avoid confusion regarding postpartum blues), were over the age of 18 years, spoke Turkish, had no significant health problems in the postpartum period, and volunteered to participate in the study were included in the study. The non-earthquake-affected group consisted of 41 mothers who were not in the earthquake zone, gave birth in Istanbul, were at least 6 weeks postpartum (to avoid confusion regarding postpartum blues), and had no significant health problems in the postpartum period.

### Data collection

The data were obtained through face-to-face interviews with women who were referred to the state hospitals where the study was conducted during the earthquake period, who gave birth in these hospitals, and who applied to these hospitals for routine control of their infants after birth. In the study, the participants were informed and asked to fill in the "Personal Information Form," "Edinburgh Postpartum Depression Scale (EPDS)," and "Traumatic Stress Symptom Checklist (TSSC)."

Personal Information Form: This form consisted of 25 questions about the sociodemographic, obstetric, and postpartum characteristics of the participants.

Edinburgh Postpartum Depression Scale (EPDS): It was developed by Cox et al., and its Turkish validity and reliability were performed by Engindeniz et al^6,7^. The responses to the 4-point Likert-type scale consist of a total of 10 questions are scored between 0 and 3. If the score obtained is 12 and below, the patient is not at risk for postpartum depression, and if the score is 13 and above, the patient is considered to be at risk for postpartum depression^7^.

Traumatic Stress Symptom Checklist (TSSC): The Turkish validity and reliability study was carried out by Başoğlu et al.^8^. The scale evaluates PTSD (according to DSM-IV) symptoms. The scale consists of three subdimensions and 17 items. A total score of 25 or above indicates PTSD^8^.

### Ethical considerations

For the study, ethical approval (IRB: 2023/129) was obtained from the ethics committee of a hospital affiliated with the Ministry of Health, study permission (2023/215209717/7) was obtained for the institutions where the study was conducted, and "Informed Consent Form" approval in accordance with the Declaration of Helsinki was obtained from the participants.

### Data analysis

The data obtained in the study were analyzed using SPSS for Windows 22.0 program. Descriptive statistical methods (number, percentage, mean, and standard deviation), student's t-test, chi-square test, and multiple linear regression analysis techniques were used to evaluate the data. In all analyses, p<0.05 was considered statistically significant.

## RESULTS

In the study, it was found that mothers who were affected by the earthquake had significantly lower levels of education (less than 8 years) and were affected by more chronic diseases than those who were not affected by the earthquake. On the other hand, the rate of unemployed women was found to be significantly higher among women who were not affected by the earthquake (p<0.05) (Table 1).

**Table 1:** Comparison of participants’ demographic and obstetric characteristics (n=82).

Variable	Earthquake-affected mothers (n:41)		Non-earthquake-affected mothers (n:41)		T	p-value
	Mean±SD		Mean±SD			
Maternal age (years)	27.63±5.62		26.58±4.57		0.927	0.357
Number of miscarriages	1.33±0.65		2.18±1.83		-1.453	0.171
Number of curettages	1.30±0.63		1.00±0.00		0.470	0.647
Number of births	2.14±1.08		2.07±1.21		0.288	0.774
Number of living children	2.12±1.14		1.97±1.19		0.567	0.573
Week of birth	37.51±1.58		39.12±1.53		**-4.673**	**0.000**
Newborn's birthweight	3,031.46±426.53		3,289.14±473.08		**-2.590**	**0.011**
**Education level**	**N**	**%**	**n**	**%**	χ^2^	**p**
≤8 years and below	25	61.0	4	9.8	21.6340*	0.000
>8 years and above	16	39.0	37	90.2		
**Employment status**						
Working	19	46.3	6	14.6	**8.286** *	**0.004**
Not working	22	53.7	35	85.4		
**Income level**						
Income is less than expenses	39	95.1	40	97.7	**	1.000
Income is equal to expenses	2	4.9	1	2.4		
**Family type**						
Nuclear family	26	63.4	26	63.4	**	1.000
Extended family	15	36.6	15	36.6		
**Chronic disease**						
Yes	9	22.0	13	31.7	**5.581** *	**0.018**
No	32	78.0	28	68.3		
**Continuous medication use**						
Yes	15	36.6	13	31.7	0.054*	0.816
No	26	63.4	28	68.3		
**Smoking status**						
Yes	5	12.2	4	9.8	**	1.000
No	36	87.8	37	90.2		
**Having any problems in the current pregnancy**						
Yes	9	22.0	1	2.4	**5.581** *	**0.018**
No	32	78.0	40	97.2		
**Regular antenatal follow-up during pregnancy**						
Yes	26	63.4	41	100.0	**15.192** *	**0.000**
No	15	36.6	–	–		
**Experiencing birth trauma**						
Yes	8	19.5	–	–	**	**0.005**
No	33	80.5	41	100.0		
**Breastfeeding status**						
Yes	34	82.9	41	100.0	**	**0.012**
No	7	17.1	–	–		

* Chi-square with Yates correction;

** Fisher's exact test.

SD: standard deviation.

Bold: p<0.05.

It was determined that earthquake-affected mothers gave birth at an average of 37.51±1.58 gestational weeks, newborns had a mean birth weight of 3,031.46±426.53 g, gave birth at a significantly earlier gestational age than mothers who were not affected by the earthquake, and their infants were found to have lower birth weights (p<0.05) (Table 1). In the study, it was found that 26.8% of the earthquake-affected pregnant women gave birth normally and 73.2% of them had cesarean section, whereas 97.6% of the earthquake-unaffected pregnant women gave birth normally and 2.4% of them had planned cesarean section due to preeclampsia.

It was determined that 22% of the earthquake-affected mothers had problems in their current pregnancies, 36.6% did not have regular prenatal follow-up, and 19.5% experienced birth trauma (Table 1).

In this study, it was determined that earthquake-affected mothers had significantly higher scores on the total and subdimensions of the TSSC compared to mothers who were not affected by the earthquake. Similarly, EPDS mean scores of earthquake-affected were found to be significantly higher than non-earthquake-affected mothers. It was found that earthquake-affected mothers showed more stress symptoms (>25) according to the TSSC cut-off score and more depressive symptoms (>13) according to the EPDS cut-off score (p<0.05) (Table 2).

**Table 2:** Comparison of participants’ Traumatic Stress Symptom Checklist and Edinburgh Postpartum Depression Scale mean scores (n=82).

Variable	Earthquake-affected mothers (n:41)		Non-earthquake-affected mothers (n:41)		t	p-value
	Mean±SD		Mean±SD			
Hypervigilance	14.51±0.59		1.58±0.99		**71.111**	**0.000**
Avoidance/emotional blunting	18.58±1.76		2.39±0.89		**52.560**	**0.000**
Reliving	13.90±0.96		2.60±1.22		**46.346**	**0.000**
TSSC total score	47.00±2.42		6.58±1.89		**83.965**	**0.000**
EPDS total score	14.92±7.15		6.46±2.54		**7.139**	**0.000**
**PTSD status**	**N**	**%**	**n**	**%**	**χ^2^**	**p-value**
25 points and below	–	–	41	100.0	**78.049** *	**0.018**
26 points and above	41	100.0	–	–		
**Postpartum depression status**						
12 points and below	11	26.8	40	97.6	**40.663** *	**0.000**
13 points and above	30	73.2	1	2.4		

* Chi-square with Yates correction.

PTSD: post-traumatic stress disorder; TSSC: Traumatic Stress Symptom Checklist, EPDS: Edinburgh Postpartum Depression Scale; SD: standard deviation. Bold: p<0.05.

In multivariate regression analysis, 43% of the variance in TSSC was found to be associated with EPDS score (beta=0.500, p<0.05) and gestational week (beta=-0.262, p<0.05) and both depression level and gestational week were found to be determinants of posttraumatic stress level in women affected by the earthquake (Table 3).

**Table 3:** Factors affecting participants’ posttraumatic stress level according to linear regression analysis.

Variables	B	Sth. error	Beta (β)	t	p	95%CI (OR)	
Constant	128.254	43.717		2.934	0.004	41.237	215.271
EPDS	1.496	0.283	0.500	5.295	**0.000**	0.934	2.059
Gestational week	-3.066	1.103	-0.262	-2.779	**0.007**	-5.261	-0.870

R: 0.659; Adjusted R^2^: 0.434; F: 30.287; p: 0.000. EPDS: Edinburgh Postpartum Depression Scale; CI: confidence interval; OR: odds ratio. Bold: p<0.05.

## DISCUSSION

The 7.7-magnitude Kahramanmaras earthquake, which is classified as an earthquake with large and devastating consequences in the world, caused much destruction and loss in 10 cities^9^. It was determined that approximately 356,000 mothers who survived the earthquake that took place in Turkey and devastated Syria needed access to emergency reproductive health services^10^. Regular antenatal follow-ups were interrupted during the devastating earthquake. With the devastation of hospitals and medical centers, pregnant women had to travel long distances to reach the nearest health institution. In addition, psychological trauma and stress, including fear of aftershocks, displacement, and loss of loved ones, made it difficult for pregnant women to receive antenatal services^11^. Similarly, as one of the significant results in this study, it was determined that mothers who were earthquake-affected had more problems during pregnancy than those who were not, and regular antenatal follow-ups could not be performed.

Exposure to earthquakes during pregnancy is an important source of stress^12^. Prenatal stress stimulates the release of cortisol and norepinephrine, which are stress hormones. Stress hormones transitioned to the fetus via the placenta constitute the biological basis of many adverse birth outcomes^12-14^. As a result of the meta-analysis study, it was reported that prenatal stressful life events are associated with a 20% higher risk of preterm birth, a 23% higher low birth weight (LBW), and a 14% higher small for gestational age (SGA)^15^. In a retrospective study with pregnant women in Iran, it was determined that the rates of preterm birth (18.91%; 10.90%), miscarriage (17.11%; 10.54%), and stillbirth (3.78%; 1.82%) were higher in pregnant women who were earthquake-affected compared to pregnant women who were not^16^. In a study that included 73,493 pregnant women in China, it was determined that the rates of stillbirth (1.33%; 2%), preterm birth (14.14%; 7.32%), LBW (10.82%; 1.33%), and SGA (11.32%; 9.52%) were higher in pregnant women who were earthquake-affected compared to pregnant women who were not^17^. Similar to the results of the study, it was found in this study that earthquake-affected mothers gave birth at an earlier gestational week and had LBW children. In this study, the threat of preterm birth was found to be a determining factor in increasing the level of posttraumatic stress, and this result supports the results of studies conducted in Iran and China.

Perinatal mental health problems due to exposure to natural disasters negatively affect the nutritional status of infants in the postnatal period. These psychological problems can cause a decrease in the amount of breast milk and interruption of breastfeeding^18,19^. It is recommended by the World Health Organization and UNICEF to continue breastfeeding during natural disasters^20^. Similar to the results of this study, unfortunately, there is a decrease in breastfeeding rates in emergencies and a corresponding increase in formula feeding^21,22^. In addition to psychological problems, the lack of suitable areas for breastfeeding due to the damage or destruction of the buildings and the collapse of health institutions that can be admitted for breastfeeding problems^21,22^.

Exposure to natural disasters during pregnancy can lead to a range of psychological problems in pregnant women, including posttraumatic stress disorder^21^. In addition to posttraumatic stress disorder, pregnant women affected by earthquakes are at increased risk of postpartum depression in the postpartum period^21,22^.

In a study of 558 earthquake survivors, 24% of mothers were found to have TSSC symptoms^21^. In another study, the rates of TSSC and postpartum depression were found to be 19.9 and 29%, respectively, in earthquake-affected mothers. The rate of TSSC and postpartum depression was found to be significantly higher in earthquake-affected mothers compared to mothers not affected by the earthquake^22^. In the cohort study conducted in Japan, the prevalence of postpartum depression was 13.3%, and the earthquake was associated with trauma and postpartum depression^22^. As a result of this study, similar to the results of other studies, it was determined that the TSSC and EPDS scores of earthquake-affected mothers were significantly higher than those of mothers who were not affected by the earthquake. However, in support of the studies conducted in this study, depressive symptoms were found to be a predictive factor for posttraumatic stress levels. It is seen that earthquake exposure in mothers causes traumatic stress, which negatively affects birth outcomes and increases the susceptibility of mothers to postpartum depression.

## CONCLUSION

In this study, it was found that women affected by the earthquake had preterm and traumatic births, their infants had LBW, and they had breastfeeding problems after birth. In addition, it was determined that all of the mothers affected by the earthquake experienced stress, and the majority of them showed depressive symptoms. Therefore, in earthquake-prone regions such as Turkey, it is extremely important to focus on women and children in the first post-earthquake intervention.
